# Eukaryotic translation initiation factor 3B accelerates the progression of esophageal squamous cell carcinoma by activating β-catenin signaling pathway

**DOI:** 10.18632/oncotarget.9726

**Published:** 2016-05-30

**Authors:** Fengkai Xu, Cheng-Zhi Xu, Jie Gu, Xiaoming Liu, Ronghua Liu, Enyu Huang, Yunfeng Yuan, Guangyin Zhao, Jiahao Jiang, Chen Xu, Yiwei Chu, Chunlai Lu, Di Ge

**Affiliations:** ^1^ Department of Thoracic Surgery, Zhongshan Hospital, Fudan University, Shanghai, P. R. China; ^2^ Department of Otolaryngology, Shanghai Ninth People's Hospital, Shanghai Jiao Tong University School of Medicine, Shanghai, P. R. China; ^3^ Department of Immunology and Key Laboratory of Medical Molecular Virology of MOE/MOH, School of Basic Medical Sciences, Fudan University, Shanghai, P. R. China; ^4^ Department of Pathology, Zhongshan Hospital, Fudan University, Shanghai, P. R. China

**Keywords:** esophageal squamous cell carcinoma, eukaryotic translation initiation factors 3B, β-catenin signaling pathway

## Abstract

**Introduction:**

Esophageal squamous cell carcinoma (ESCC) is one of the most aggressive malignant tumors. Eukaryotic translation initiation factors 3B (EIF3B) is considered to influence tumor proliferation, invasion, apoptosis and cell cycle, which act together to promote the progression of tumors. However, the role of EIF3B in ESCC is unknown. This study aims to explore the clinical and biological role of EIF3B in ESCC.

**Results:**

EIF3B expressions were up-regulated in both ESCC tissues and cell lines. Overexpression of EIF3B was associated with tumor depth, lymph node metastasis and advanced TNM stage. Importantly, patients with high EIF3B expression suffered shorter overall and disease-free survival. Knockdown of EIF3B could inhibit cell proliferation and invasion, promote cell apoptosis, and interfere the cell cycle *in vitro*. EIF3B-knockdown cells could form smaller subcutaneous tumors *in vivo*. Finally, we demonstrated EIF3B could activate β-catenin signaling pathway.

**Methods:**

Immunohistochemical staining and Western blot were performed to detect the EIF3B expression in ESCC patient tissues and cell lines. The association between EIF3B expression and patients’ prognosis was analyzed by Kaplan-Meier and Cox regression. Then, CCK-8, colony-formation, Transwell and wound-healing assay were performed to compare the bio-functional change after knockdown of EIF3B. Flow cytometry was applied to analyze the change of cell apoptosis and cycle induced by EIF3B knockdown. Tumor xenograft assay was done to verify the *in-vitro* results.

**Conclusions:**

EIF3B might serve as a novel marker for predicting prognosis of ESCC patients and as a potential therapeutic target, individually or together with other subunits of EIF3 complex.

## INTRODUCTION

Esophageal squamous cell carcinoma (ESCC) is one of the most aggressive malignant tumors throughout the world. In China, the incidence of ESCC greatly outnumbers that of esophageal adenocarcinoma [1]. In spite of the recent improvements achieved in diagnostic and therapeutic methods, no significant improvements have been achieved in the 5-year survival rate due to high rate of early metastasis [2, 3]. Thus, it is quite urgent to discover the mechanism deeply under the initiation and progression of ESCC so as to gain new therapeutic strategies for ESCC.

The progression of ESCC could be influenced by numerous factors, among which protein synthesis plays an important role. Meanwhile, the control of translation is one of the important regulation points, which is the early step in the protein synthesis. Eukaryotic translation initiation factors (EIF) are the indispensable components for the formation of an initiation complex that participate in assembling the 80S-mRNA-Met-tRNA complex to initiate the protein synthesis [4]. Among the family of EIF, EIF3 is the largest factor and composed of 13 subunits: A-M, contributing to the maintenance of 40S subunits and assembling of 43S pre initiation complex. In different solid malignant tumors, different EIF3 subunits have been reported to show shift expression [5, 6]. Eukaryotic translation initiation factors 3B (EIF3B), serving as a scaffolding subunit, has been verified to be overexpressed in colon cancer, bladder and prostate cancer, glioblastoma, etc. and also made difference in the biological characteristics of cancer cells [7–9]. However, no related research has been done in ESCC.

In this study, we demonstrated that EIF3B expression was up-regulated in ESCC cells and negatively correlated with the prognosis of patients. Knock-down of EIF3B expression with small interfering RNA (siRNA) could significantly inhibit the progression of ESCC cells both *in vitro* and *in vivo*. EIF3B accelerated the progression of ESCC via the activation of β-catenin signaling pathway.

## RESULTS

### EIF3B is overexpressed in ESCC and negatively correlated with the prognosis

**Figure 1:**
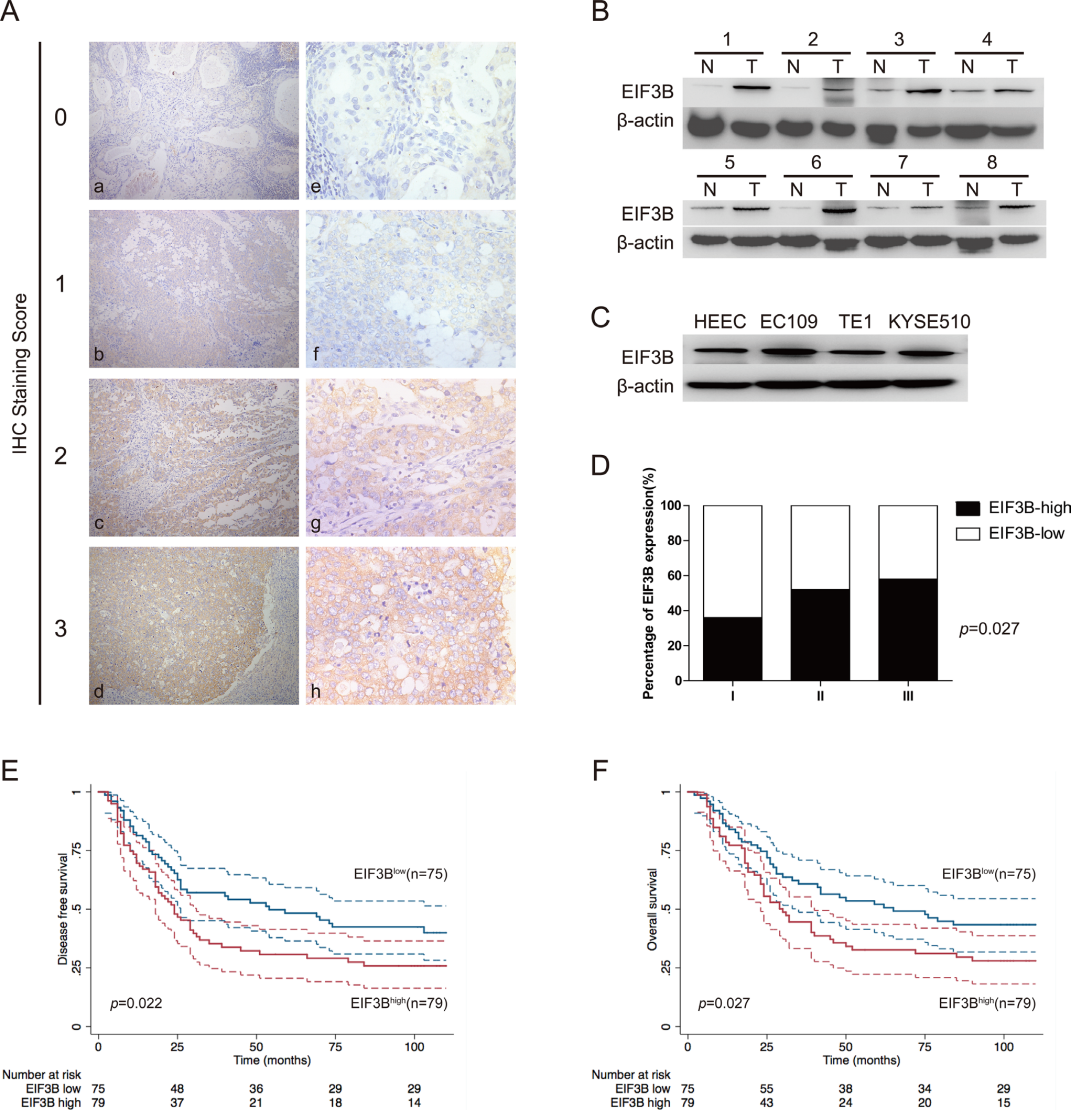
EIF3B expression is overexpressed in ESCC and negatively associated with patents’ prognosis (**A**), IHC (× 200 for a–e and × 400 for e–h) analysis of EIF3B expression in ESCC tissues from 154 enrolled patients. Typical images of EIF3B expression level from score 0 to 3 were exhibited. (**B** and **C**), Western blot assay was performed to detect the EIF3b expression in ESCC tumors, corresponding para-cancerous tissues and cell lines. β-actin was used as an internal reference. (**D**), percentage of EIF3B expression level in each histological grade. (**E** and **F**), DFS and OS curves of 154 enrolled ESCC patients, obtained by Kaplan-Meier product limit method, were compared according to EIF3B expression level. The plain lines are the mean value of the survival. The range between the dashed lines are the confidence interval of the survival.

In the 154 ESCC tissue samples, we applied the IHC to detect the expression of EIF3B and found that EIF3B expression was nearly undetectable in normal epithelium of esophagus. However, EIF3B exhibited cytoplasmic staining in ESCC tissues (Figure 1A). According to the evaluation criteria described in the method, the number of cases with low (score 0, 1) and high (score 2, 3) expression of EIF3B was 75 and 79, respectively. Next, we performed Western blot assay to detect the expression of EIF3B in 8 pairs of fresh primary ESCC tumors and corresponding para-cancerous tissues. We observed that the EIF3B expression in ESCC tissues was significantly higher than that in para-cancerous ones (Figure 1B). Moreover, we investigated the EIF3B protein level in one human normal esophageal epithelial cell line (HEEC) and three ESCC cell lines (EC109, TE1 and KYSE510). As shown in Figure 1C, the EIF3B expression in EC109 and KYSE510 was higher than that in HEEC (Figure 1C and Supplementary Figure 1). Therefore, we picked up EC109 and KYSE510 for further bio-functional studies.

To investigate the correlations between EIF3B expression and clinicopathological features, we employed statistical analysis and found that high expression of EIF3B was positively associated with tumor depth, lymph node metastasis and TNM stage, significantly (*P* < 0.05) (Table 1). EIF3B expression was also elevated with the up-grade of tumor histological differentiation level, although the difference did not reach the criterion of significance (Figure 1D), which indicated that high expression of EIF3B was positively correlated with the poor differentiation of tumor. During the follow-up, recurrence or metastasis occurred in 99 patients and the metastatic areas included supraclavicular lymph node, mediastinal lymph node, liver, lung, skeleton and brain. Besides, 95 patients were died of ESCC. In univariate analysis, patients with high EIF3B expression suffered low DFS and OS compared with the ones with low EIF3B expression (*P* < 0.05). Graphic pattern of Kaplan-Meier curves suggested that prognosis was poor for patients with high EIF3B expression (Figure 1E and 1F). Tumor depth, lymph node metastasis and TNM stage were significantly correlated with patients’ DFS and OS (*p* < 0.01) (Table 2). In multivariate analysis, “lymph node metastasis” was identified as an independent factors in patients’ prognosis (Table 3).

**Table 1:** Analysis of the associations between EIF3B expression and clinicopathologic features

Clinicopathologic features		EIF3B expression		*P* value
		Low	High	
**Total**	154	75 (49)	79 (51)	
**Age**				
≤ 60 years	76	38 (50)	38 (50)	0.751
> 60 years	78	37 (47)	41 (53)	
**Sex**				
Male	119	56 (47)	63 (53)	0.454
Female	35	19 (54)	16 (46)	
**Tumor size (cm)**				
< 3	69	36 (52)	33 (48)	0.439
≥ 3	85	39 (46)	46 (54)	
**Tumor depth**				
T1	12	10 (83)	2 (17)	**0.017**
T2	41	23 (56)	18 (44)	
T3	95	41 (43)	54 (57)	
T4	6	1 (17)	5 (83)	
**Lymph node metastasis**				
No	91	52 (57)	39 (43)	**0.012**
Yes	63	23 (37)	40 (63)	
**TNM stage**				
I A + I B	12	10 (83)	2 (17)	**0.0026**
II A + IIB	83	45 (54)	38 (46)	
III A + III B + III C	59	20 (34)	39 (66)	
**Histological grade**				
I	14	9 (64)	5 (36)	0.410
II	114	55 (48)	59 (52)	
III	26	11 (42)	15 (58)	

a Bold values indicate statistical significance (*p* < 0.05).

Abbreviation: ESCC, Esophageal squamous cell carcinoma.

**Table 2:** Univariate analysis of ESCC patients’ survival

Variables	DFS			OS		
	Events/patients (%)	Median DFS (mo)	*P* value	Events/patients (%)	Median OS (mo)	*P* value
**Age**						
**≤ 60 years**	43/76 (57)	39	0.067	40/76 (53)	52	**0.020**
**> 60 years**	56/78 (72)	26		55/78 (71)	28	
**Sex**						
**Male**	80/119 (67)	26	0.100	77/119 (65)	31	0.062
**Female**	19/35 (54)	74		18/35 (51)	84	
**Tumor size**						
**< 3cm**	43/69 (62)	30	0.433	40/69 (58)	46	0.272
**≥ 3cm**	56/85 (66)	26		55/85 (65)	39	
**Tumor depth**						
**T1**	4/12 (33)	NR	**0.002**	1/12 (8)	NR	**0.000**
**T2**	23/41 (56)	66		22/41 (54)	76	
**T3**	67/95 (71)	22		67/95 (71)	29	
**T4**	5/6 (83)	11		5/6 (83)	11	
**Lymph node metastasis**						
**No**	43/91 (47)	103	**0.000**	42/91 (46)	NR	**0.000**
**Yes**	56/63 (89)	18		53/63 (84)	24	
**TNM stage**						
**I A + I B**	2/12 (17)	NR		1/12 (8)	NR	
**II A + II B**	45/83 (54)	66	**0.000**	42/83 (51)	79	**0.000**
**III A + III B + III C**	52/59 (88)	16		52/59 (88)	23	
**Histological grade**						
**I**	5/14 (36)	NR	0.071	4/14 (29)	NR	**0.048**
**II**	77/114 (68)	26		75/114 (66)	35	
**III**	17/26 (65)	24		16/26 (62)	28	
**EIF3B expression**						
**Low**	43/75 (57)	54	**0.022**	41/75 (55)	65	**0.027**
**High**	56/79 (71)	24		54/79 (68)	30	
**Postoperative chemotherapy**						
**No**	51/87 (59)	45	0.092	48/87 (55)	52	0.109
**Yes**	48/67 (72)	24		47/67 (70)	30	

a Bold values indicate statistical significance (*p* < 0.05).

Abbreviation: ESCC, Esophageal squamous cell carcinoma; EIF3B, Eukaryotic translation initiation factors; NR, not reached.

**Table 3:** Multivariate analysis of ESCC patients’ survival

Variables	DFS			OS		
	HR	95% CI	*P* value	HR	95% CI	*P* value
**Age**	1.564	0.983 ~ 2.488	0.059	1.853	1.143 ~ 3.004	**0.012**
**Sex**	1.066	0.633 ~ 1.795	0.810	1.129	0.664 ~ 1.917	0.654
**Tumor size**	0.900	0.591 ~ 1.370	0.623	0.877	0.570 ~ 1.350	0.551
**Tumor depth**	1.338	0.953 ~ 1.879	0.093	1.699	1.187 ~ 2.432	**0.004**
**Lymph node metastasis**	2.683	1.733 ~ 4.152	**0.000**	2.256	1.456 ~ 3.497	**0.000**
**Histological grade**	1.280	0.851 ~ 1.925	0.235	1.314	0.855 ~ 2.019	0.213
**EIF3B expression**	1.194	0.783 ~ 1.820	0.411	1.134	0.739 ~ 1.742	0.565
**Postoperative chemotherapy**	1.378	0.861 ~ 2.206	0.181	1.414	0.873 ~ 2.290	0.159

a Bold values indicate statistical significance (*p* < 0.05).

Abbreviation: ESCC, Esophageal squamous cell carcinoma; DFS, Disease-free survival; OS, overall survival; HR, hazard ratio; CI, confidence interval; EIF3B, Eukaryotic translation initiation factors.

### EIF3B promotes the cell proliferation and invasion of ESCC

**Figure 2:**
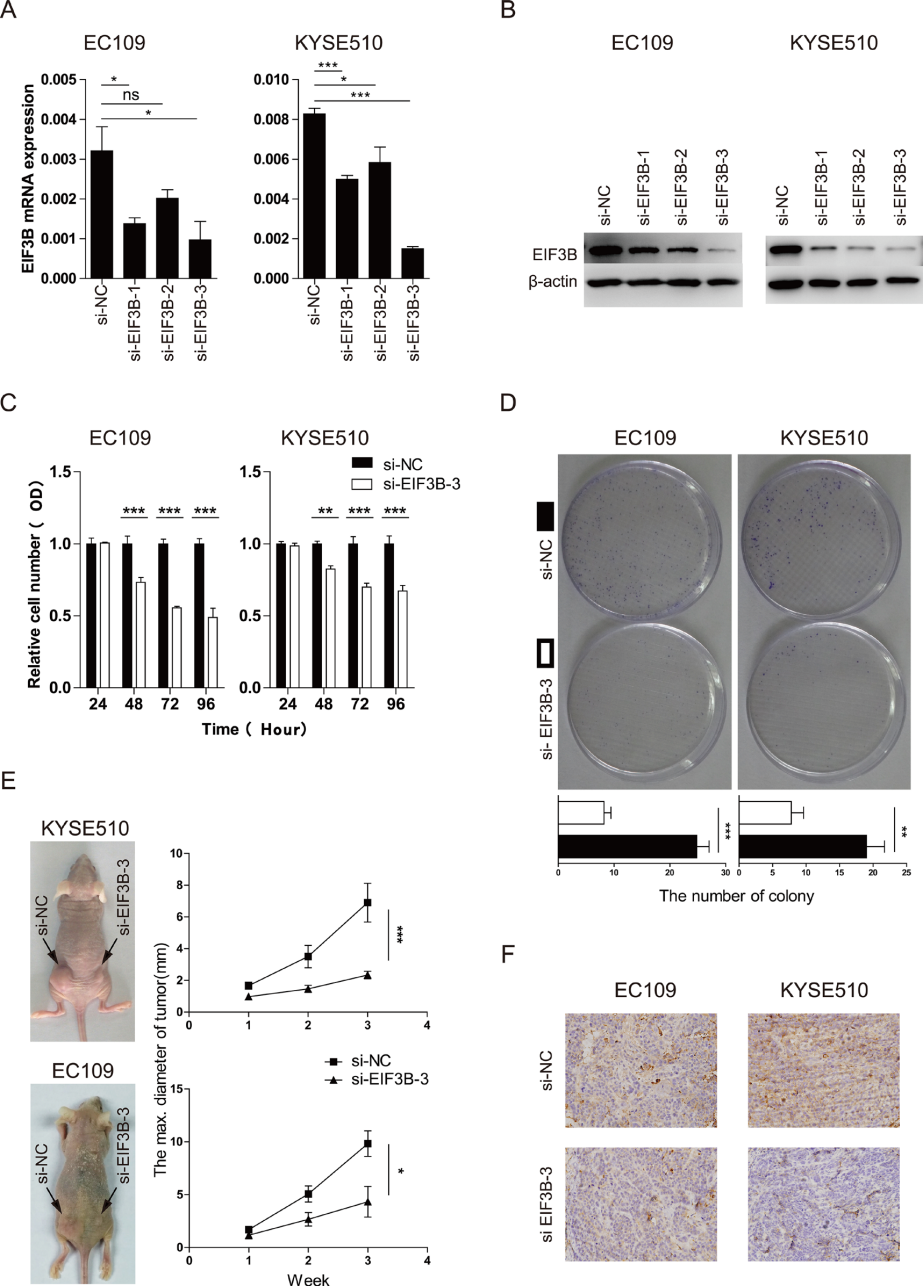
EIF3B promotes the cell proliferation of ESCC (**A** and **B**), the effect of the knockdown was validated through qRT-PCR and Western blot analyses. β-actin was used as an internal reference. (**C**), the proliferative ability was assessed with CCK-8 assay at 24, 48, 72, and 96 hours after transfection. (**D**), the proliferative ability was assessed with colony-forming assay and analyzed statistically after 10-day culture. (**E**), the proliferative ability was assessed with tumor xenograft assay and analyzed statistically 3 weeks after implantation. (**F**), the representative staining intensity of EIF3B expression in transplanted tumors was detected with IHC. The values were shown as the mean ± SD. (ns: no significance, *p <0.05, **p <0.01, ***p < 0.001).

To explore the importance of EIF3B expression for the progression of ESCC further, we constructed 3 pairs of siRNA to knock down the EIF3B expression. The efficiency of the transfection was high according to the Cy3-modified expression under light microscope and fluorescence microscope (Supplementary Figure 2). The effect of the knockdown was validated through Western blot and qRT-PCR analyses. As shown in the Figure 2A and 2B, the EIF3B-siRNA-3 showed the best effect of knockdown and thus picked up for further study.

We applied CCK-8 and colony-forming assay to study the proliferative change after knockdown of EIF3B and found that, compared with the normal control (NC) groups, both two cell lines with knockdown of EIF3B showed low proliferative ability, which indicated that EIF3B promoted the proliferation of ESCC (Figure 2C and 2D). In addition, *in vivo*, tumor xenograft assay was performed to verify that EIF3B made a difference in the proliferation ability. The results showed that NC groups could form significantly larger tumors than the EIF3B knockdown groups did, which was consistent to the previous results *in vitro* (Figure 2E). Furthermore, we applied IHC assay to detect the EIF3B expression in each sample and found that EIF3B expressions were higher in NC group than those in knockdown group (Figure 2F and Supplementary Table 1).

**Figure 3:**
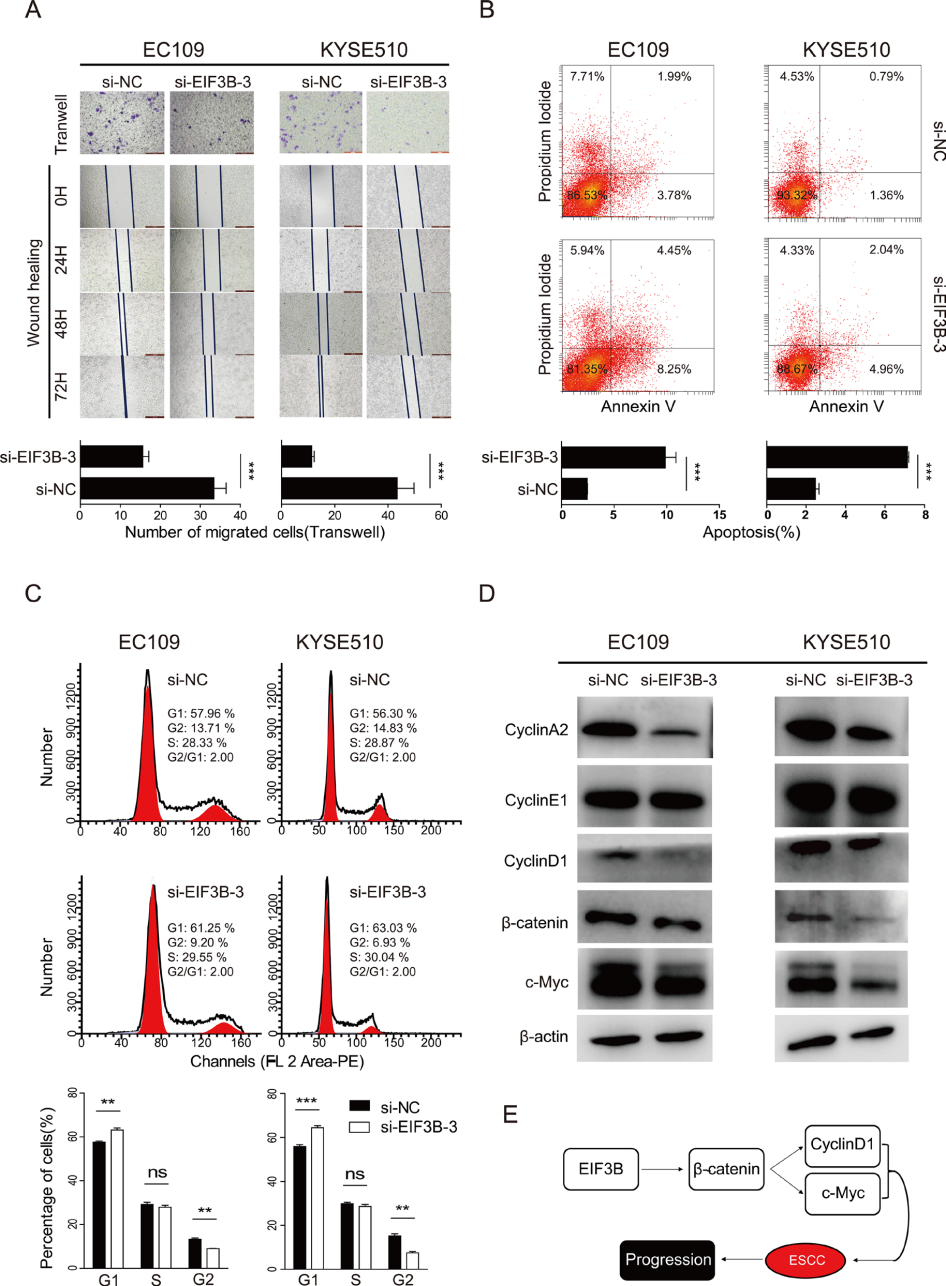
EIF3B promotes the cell invasion, inhibits the cell invasion and interfere the cell cycle of ESCC by activating the β-catenin signaling pathway (**A**), the invasion ability was assessed with Transwell and wound-healing assay. (**B**), the apoptosis of cells was detected with flow cytometry after staining of Annexin V and PI. (**C**), the cell cycle was analyzed with flow cytometry after staining of PI. The values were shown as the mean ± SD. (**D**), Western blot was performed to analyze the shift of related protein expression level after knockdown of EIF3B. β-actin was used as an internal reference. (**E**), graph summarized the mechanism for EIF3B to accelerate the progression of ESCC. (ns: no significance, *p < 0.05, **p < 0.01, ***p < 0.001).

Next, we performed Transwell and wound-healing assay to study the role of EIF3B in the cell invasion of ESCC. The results both showed that a significant decrease in cell invasion in both two cell lines transfected with EIF3B-siRNA-3 compared with NC groups (Figure 3A). Collectively, our results demonstrated that EIF3B played a vital accelerating role in the promotion of cell proliferation and invasion.

### EIF3B inhibits the cell apoptosis and interfere the cell cycle of ESCC

It has been reported that EIF3B influence the cell apoptosis and cell cycle in some solid tumors [7, 9]. We tried exploring whether similar effects of EIF3B could be found in ESCC. For the analysis of apoptosis, cells were stained with Annexin V and PI sequentially, then analyzed with FACS. The results showed that the total apoptosis percentages of knockdown groups were significantly larger than those of NC groups in each cell line (Figure 3B, *p* < 0.05), which indicated that EIF3B inhibited the cell apoptosis of ESCC. In the detection of apoptotic change of mice tumors, we applied TUNEL assay. The samples of each subgroup were incubated with the TUNEL reagent and DAPI. The fluorography showed that the apoptosis levels of knockdown groups were significantly higher than those of NC groups in each cell line (Supplementary Figure 3, *p* < 0.05).

For the analysis of cell cycle, we stained the two cell lines with PI according to the protocol and analyzed them with FACS. The results showed that knockdown of EIF3B expression significantly decreased the percentage of cells in G2 phase, increased the percentage of cells in G1 phase, and the percentage of cells in S phase almost showed no significant difference, which implied the existence of EIF3B made difference in the cell cycle (Figure 3C). Then, we tried to explore the potential mechanisms and applied Western blot assay to detect the shift of related influential factors expression. The results showed that Cyclin A and D1 expression was down-regulated after the knockdown of EIF3B, while Cyclin E expression remains almost the same (Figure 3D). These data was in consistent to the former phenomena that EIF3B stimulated both S and G2 phase entry. After knockdown of EIF3B, S and G2 phase entry were inhibited. S phase inhibition led to more cells staying in G1 phase but less cells entering S phase. G2 phase inhibition led to more cells staying in S phase but less cells entering G2 phase. Therefore, it is reasonable that after knockdown of EIF3B, cells in G1 phase were increased, in G2 phase were decreased and in S phase were almost unchanged.

### EIF3B activates β-catenin signaling pathway

It has been reported that β-catenin signaling pathway is one of most frequent pathway involved in the initiation and progression of solid malignant tumors [10]. On the other hand, EIF3i, another subunit of EIF3 complex has been demonstrated to activate β-catenin pathway [11]. Thus, we speculated whether EIF3B accelerate the progression of ESCC through β-catenin pathway. The previous Western blot results showed that knockdown of EIF3B down-regulated Cyclin D1, a β-catenin target gene [12], expression in both two cell lines. Next, we detected β-catenin and another well-known β-catenin target gene, c-Myc [13], protein level and found these two gene could be also down-regulated consistently with Cyclin D1 (Figure 3D). Besides, we applied qRT-PCR analysis to detect the mRNA level change of the above gene and found that all the gene were down-regulated at mRNA level after knockdown of EIF3B (Supplementary Figure 4). Taken together, our data demonstrated that EIF3B could activate β-catenin pathway to accelerate the progression of ESCC (Figure 3E).

## DISCUSSION

EIF3B has been presented as an important scaffold subunit for EIF3B complex, which plays a decisive and accelerating role in the initiation of translation [14]. It has been reported that EIF3B is overexpressed in several solid malignant tumors [7–9]. However, the role of EIF3B in ESCC remains unknown. In this study, we demonstrated that EIF3B was overexpressed in ESCC cell lines, compared with normal human esophageal epithelial cell line, and cancerous tissues compared with the normal para-cancerous tissues. In clinical features, tumor depth, lymph node metastasis and TNM stage were positively correlated with EIF3B expression, significantly (*P* < 0.05) (Table 1). Through the survival analysis, data indicated that patients with high EIF3B expression suffered shorter DFS and OS. In order to investigate the biological role of EIF3B, we verified that knockdown of EIF3B expression could promote cell proliferation and invasion, inhibit cell apoptosis, and interfere the cell cycle, which act together to accelerate the progression of ESCC.

The bio-functional results were almost in consistent to the previous studies done in other malignant tumors, except the way of interference of cell cycle. Liang et al. [9] revealed that knockdown of EIF3B could inhibit the G1-S transition in glioblastoma cell lines. However, Wang et al. [7] have found the opposite results that knockdown of EIF3B could facilitate the G1-S transition in colon cancer cells. These indicated that some conflicts exist in the role of EIF3B in the cell cycle regulation in different tumors. In this study, our data also showed that the existence of EIF3B made difference in the cell cycle. In the analysis of mechanism, we found that Cyclin A and D1 expression was down-regulated after the knockdown of EIF3B, while Cyclin E expression remains almost the same (Figure 3D). As Cyclin A is one of the positive regulatory protein for the G2 phase entry and Cyclin D1 for S phase entry [15], after knockdown of EIF3B, S and G2 phase entry were inhibited. S phase inhibition led to more cells staying in G1 phase but less cells entering S phase. G2 phase inhibition led to more cells staying in S phase but less cells entering G2 phase. Therefore, down-regulation of Cyclin A and D1 after the knockdown of EIF3B is consistent to the cell cycle change that cells in G1 phase were increased, in G2 phase were decreased and in S phase were almost unchanged (Figure 3C). Since the cell cycle was adjusted by numerous factors [16], the deeply mechanisms behind the regulation of cell cycle needs further exploration. These conflicts suggested that the effect of EIF3B expression on cell cycle might be specific to cancer type.

The exact pathway for EIF3B to accelerate the malignant tumor progression has not been announced. Wang et al. [8] have studied the role of EIF3B in bladder and prostate cancer and found that EIF3B could promote tumor progression through up-regulation of integrin α5. We tried to explore the further potential pathway and convert our sights to other EIF3 subunits, which might act together with EIF3B. As mentioned in the Wang et al. [8] data, both EIF3c and EIF3i expressions were up-regulated in cancer cells than normal ones. They also verified that the bio-functional changes caused by EIF3c depletion match those observed with eIF3B depletion. On the other hand, EIF3a was also reported to be up-regulated in urinary bladder cancer [17] and acted with HER-2 in ovarian cancer [18]. We chose to fix our eyes on EIF3i and previous study has showed that EIF3i promote the colon oncogenesis through β-catenin activation [11]. On the other hand, β-catenin signaling pathway has been presented as a classic pathway for the progression of different malignant tumors, including ESCC [10, 19]. Therefore, we assumed that EIF3B might act in the similar way and performed the corresponding assays. Our data showed that knockdown of EIF3B could down-regulate β-catenin level and its downstream target gene, Cyclin D1 and c-Myc, protein levels in both two cell lines. These results indicated that EIF3B could activate the β-catenin signaling pathway, which lead to the progression of ESCC. Further study should be paid into the other subunits and contributes to drawing an overall picture of the role of EIF3 complex in the progression of EIF3B.

To conclude, we have demonstrated that EIF3B was overexpressed both in ESCC tissues and cell lines. Patients with high EIF3B expression suffered poor prognosis. EIF3B could activate the β-catenin signaling pathway, including the downstream target gene Cyclin D1 and c-Myc, to induce ESCC cells proliferation and invasion, inhibit apoptosis and interfere cell cycle. Thus, EIF3B could be served as a novel marker for predicting the prognosis of ESCC patients and as a potential therapeutic target, individually or together with other subunits of EIF3 complex.

## MATERIALS AND METHODS

### Tissue samples and follow-up

Primary ESCC tissue samples were obtained from 154 patients who underwent radical esophagectomy in the Department of Thoracic Surgery, Zhongshan Hospital, Fudan University, Shanghai, China, in 2006. All sections of paraffin-embedded primary tumor tissues were reviewed by two experienced pathologists without knowledge of the patients’ information and the purpose of the research. After surgery, patients were followed up every 3 months during the first year and every 6 months thereafter. Patient data included details related to sex, date of birth, tumor pathology, tumor-node-metastasis (TNM) stage (according to American Joint Committee on Cancer, Seventh edition) [20], disease-free survival (DFS; defined as the time elapsed from the date of surgery to the discovery of local relapse or distant metastasis), and overall survival (OS; defined as the time elapsed from the date of surgery to death). 138 patients were in possession of complete information on patient survival, while 16 patients were lost in follow-up. The last date of follow-up was March 31, 2015. This study was approved by the ethics committee on human research of Zhongshan Hospital.

### Immunohistochemical staining (IHC) and evaluation

Immunohistochemical (IHC) staining was performed as previously described [21]. Then, EIF3B antibody (Abcam, Cambridge, UK) was used to detect the expression of the related protein. Based on the measurement standard described previously [22], all cases were separated into two groups: a low expression level group for scores 0 and 1, and a high expression level group for scores 2 and 3.

### Cell lines culture, transfection and Western blot assay

The human ESCC cell line EC109, TE1, and normal human esophageal epithelial cell line HEEC were cultured in DMEM, and ESCC cell line KYSE510 was maintained in RMPI 1640, all supplemented with 10% fetal bovine serum and 100 IU/ml penicillin/streptomycin in a humidified incubator under 95% air and 5% CO_2_ at 37°C.

Lipofectamine Plus reagent or Lipofectin 2000 (Invitrogen, Carlsbad, USA) was applied for transient transfection. Predesigned siRNA duplexes were purchased from Sangon Company. The sequences of siRNA-EIF3B-1 are 5′-CUGGAUACGCUUAGCAUCUdTdT-3′ (F) and 5′-AGAUGCUAAGCGUAUCCAGdTdT-3′ (R). The sequences of siRNA-EIF3B-2 are 5′-GAGAGAAGG CGCACCAUGAdTdT-3′ (F) and 5′-UCAUGGUGCGCCU UCUCUCdTdT-3′ (R). The sequences of siRNA-EIF3B-3 are 5′-GCUACAAGCUUGACAAGCAdTdT-3′ (F) and 5′- UGCUUGUCAAGCUUGUAGCdTdT-3′ (R). The knockdown group is cells transfected with siRNA-EIF3B sequence. The normal control group is cells transfected with irrelevant siRNA sequence. The transfection was performed according to the manufacturer's instructions.

For Western blot assay, the protein extraction was performed at 48 hours after transfection. The total protein were adjusted into equal amounts and subjected to SDS-PAGE. Specific antibody was used to detect the corresponding protein.

### RNA extraction and real-time qRT-PCR analysis

The total RNA extraction was performed at 24 hours after transfection. The primers of EIF3B are 5′-GGACCCGACCGACTTGAGA-3′ (F) and 5′-TTGACC CGGAATGTGTGCTG-3′ (R). The primers of β**-**actin are 5′-TGACGTGGACATCCGCAAAG-3′ (F) and 5′-CTGGA AGGTGGACAGCGAGG-3′ (R). The RNA extraction and real-time qRT-PCR were performed as described elsewhere [23].

### CCK-8 assay and colony-forming assay

CCK-8 assay was performed as previously described [24]. The time point of the proliferative-detection was 24, 48, 72, 96 hours after transfection. For colony-forming assay, 1.5 × 10^2^ cells were placed into a 60 × 15 mm cell culture dish (3 repeat wells for each subgroup). The process was applied as previously described [24]. Cells of each group were cultured for 10 days. Colonies with more than 15 cells were counted from 5 random visual fields of 5 × 5 square grids.

### Transwell and wound healing assay

For Transwell assay, at 24 hours after transfection, cells were collected. The process was applied as previously described [22]. For wound healing assay, 6-well plates were chosen for healing assay. When cells were cultured for 24 hours after transfection, a plastic pipette tip was used to scratch a line across the cell surface in each plate. The remaining cells were washed three times with PBS to remove the floating cells and debris. The images of the healing process were photographed digitally at the time point of 0, 24, 48, 72 hours after wounding.

### Facs analysis of apoptosis and cell cycle

For the analysis of apoptosis, 48 hours after transfection, cells were collected and washed three times with PBS, then suspended in 100 μl PBS, added with the 5 ul Annexin V-APC and incubated at 4°C for half an hour in the dark. 1 μl propidium iodide (PI) was added into the cell suspension and incubated at 4°C for 5 minutes in the dark. The cells stained with Annexin V and PI were detected with flow cytometry on a FACScan (BD Biosciences, NJ, USA). For the analysis of cell cycle, cells were collected at the time point of 48 hours after transfection. Cells were washed three times with PBS and fixed in 70% pre-chilled ethanol at 4°C for 1 hour. Then the cells were suspended in 500 ul PBS, added with 50 μg/ml PI and 100 μg/ml ribonuclease, incubated at 4°C for half an hour in the dark. A 300-mesh nylon net was used to filter and separate the aggregated cells. The cell cycle was analyzed with flow cytometry on a FACScan.

### Tumor xenograft assay and TUNEL assay

Female 4-week-old female nude mice were obtained from Slaccas Company. 2 × 10^6^ cells of each group, at 12 hours after transfection, were injected subcutaneously into the mice. For the same cell line, the normal control group was implanted into the left posterior flank and the knockdown group was in the right of the same mouse. The size of tumors were measured every week by caliper. 3 weeks later, the mice were executed and tumors were statistically analyzed. The apoptotic change of tumors were detected by the TUNEL staining using apoptosis detection kit (Vazyme Biotech, Nanjing, China). The tumors were made into paraffin sections and then settled according to the protocol. The samples were incubated with the TUNEL reagent containing BrightGreen Labeling Mix and Recombinant TdT Enzyme at 37°C for 1 hour. The samples were washed in 1X PBS and incubated with DAPI for 5 minutes. Fluorescence microscope at 10X magnification. The total number of both DAPI and TUNEL positive cells were counted from at least five random visual fields from each sample. Each experiment was repeated 3 times.

### Statistical analysis

Statistical analysis was performed with the Stata 11.0 software program (StataCorp LP, College Station, TX, USA). The correlations between the expression of EIF3B and clinical characteristics were assessed by the Wilcoxon (two groups) or Kruskal-Wallis (more than two groups) rank test. The Kaplan-Meier product limit method was applied to draw DFS and OS curves. The log-rank test was applied for univariate analysis. Cox proportional-hazards regression was used to analyze the effects of several risk factors on DFS and OS. The risk factors (covariates) were considered age, sex, tumor size, tumor depth, lymph node metastasis, histological grade, TNM stage, postoperative chemotherapy and EIF3B expression. The data gathered *in vitro* experiments were noted as mean ± standard deviation, and the Student's *t*-test, χ^2^ test, Fisher's exact test, and Spearman coefficients test were applied for the comparison of individual variables in the right way to analyze the difference between each subgroup. Two-tailed tests at *P* < 0.05 were considered statistically significant.

## SUPPLEMENTARY MATERIAL TABLE AND FIGURES


