# Supplementary material for: Eukaryotic translation initiation factor 3B accelerates the progression of esophageal squamous cell carcinoma by activating β-catenin signaling pathway
Source: Oncotarget. 2016 May 30;7(28):43401–11. doi: 10.18632/oncotarget.9726 (PMC5190032; doi:10.18632/oncotarget.9726)
Supplement: Supplementary file 1 [file oncotarget-07-43401-s001.pdf]

Eukaryotic translation initiation factor 3B accelerates the progression of esophageal squamous cell carcinoma by activating β-catenin signaling pathway

Supplementary Materials

Supplementary Table S1: Analysis of the EIF3B expression in xenograft tumors of nude mice

Mouse No.	EIF3B expression intensity	
	si-NC	si-EIF3B-3
EC109-1	0	1
EC109-2	1	2
EC109-3	1	2
KYSE510-1	2	3
KYSE510-2	2	3
KYSE510-3	0	2

[a]The staining intensity was scored as 0 (negative), 1 (weak), 2 (intermediate), or 3 (strong).

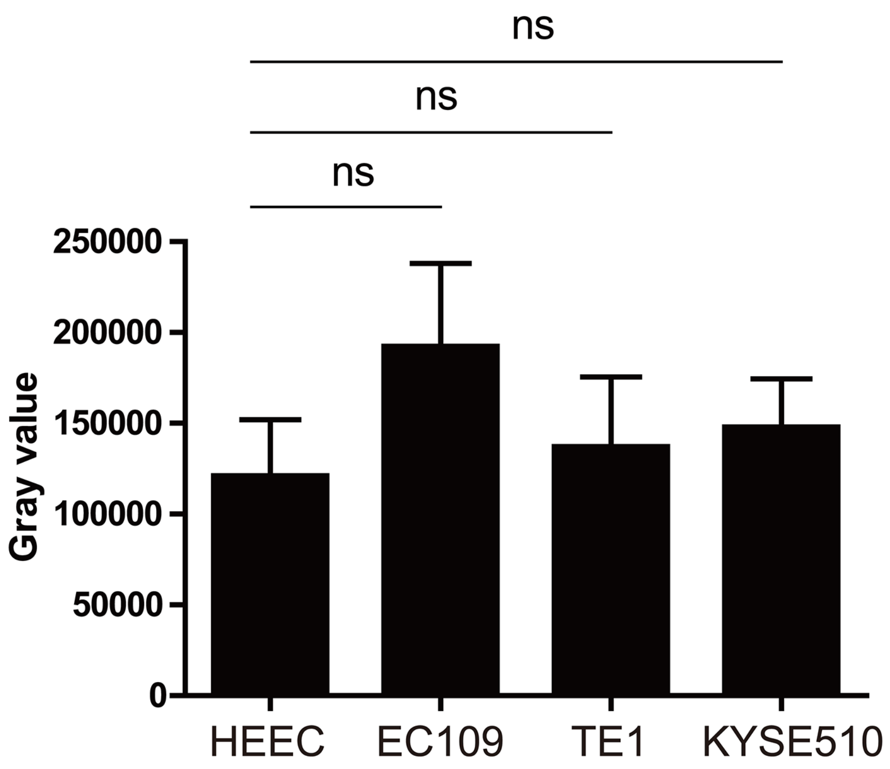

Supplementary Figure S1: The gray values of protein level of EIF3B in cell lines. The gray values of protein level of EIF3B in cell lines were analyzed by Image J. (ns: no significance, *$p < 0.05$, **$p < 0.01$, ***$p < 0.001$).

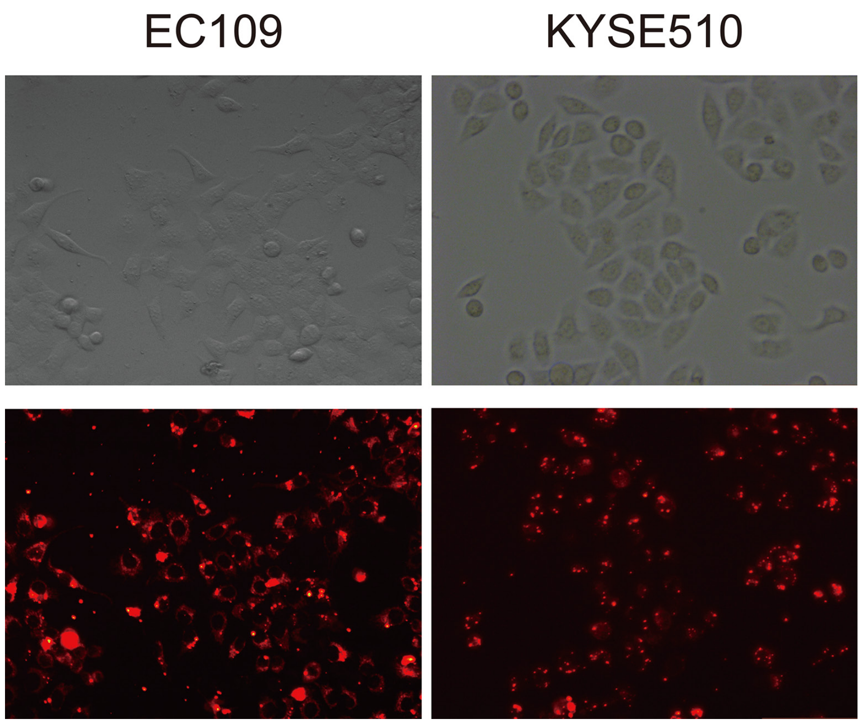

Supplementary Figure S2: The efficiency of the transfection. The efficiency of the transfection was detected according to Cy3-modified expression under light microscope (upper) and corresponding fluorescence microscope (lower) at 12 hours after transfection.

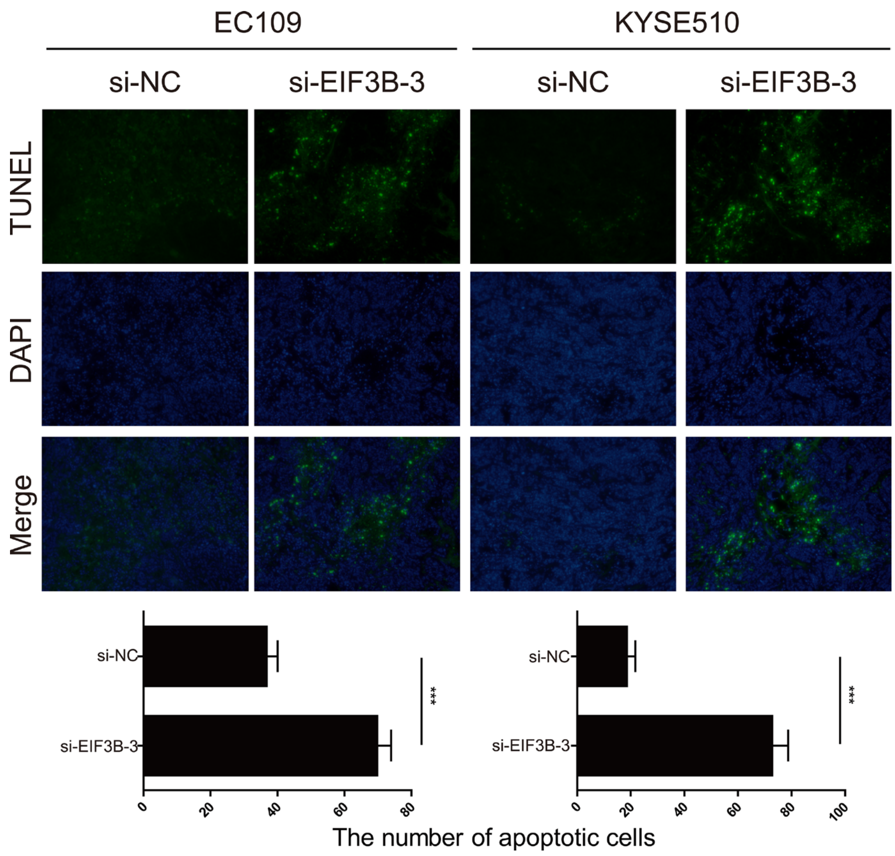

Supplementary Figure S3: The apoptosis level of implanted tumors. The apoptosis level of implanted tumors were detected by the TUNEL assay. The statistical figure below were corresponding analysis of difference of apoptosis after knockdown of EIF3B (ns: no significance, *$p < 0.05$, **$p < 0.01$, ***$p < 0.001$).

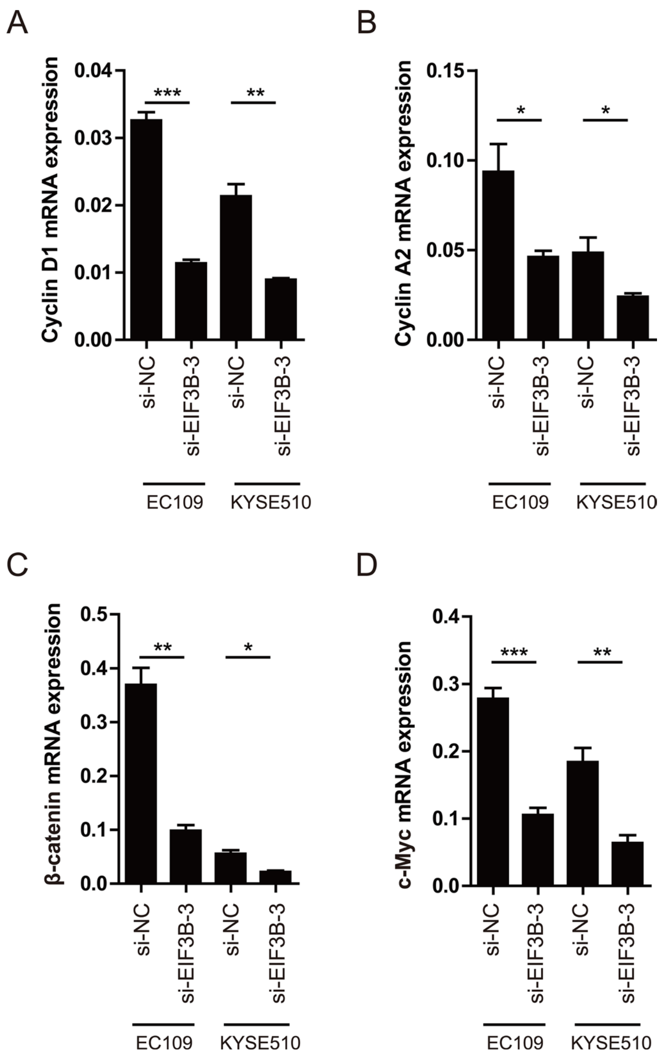

Supplementary Figure S4: Related mRNA expression level after knockdown of EIF3B. RT-PCR was performed to analyze the shift of related mRNA expression level after knockdown of EIF3B. (**A**) Cyclin D1. (**B**) Cyclin A2. (**C**) β-catenin. (**D**) c-Myc (ns: no significance, *$p < 0.05$, **$p < 0.01$, ***$p < 0.001$).
